# Intravitreal anti-VEGF therapy for extrafoveal macular neovascularisation secondary to age-related macular degeneration: five-year results in a tertiary centre

**DOI:** 10.1038/s41433-025-04057-w

**Published:** 2025-11-06

**Authors:** Faik Gelisken, Nurullah Koçak, Caroline J. Wenzel, Kübra Atay Dinçer, Daniel A. Wenzel

**Affiliations:** 1 Department of Ophthalmology, University of Tübingen, Tübingen, Germany; 2 Department of Ophthalmology, Ondokuz Mayis Universitesi, Samsun, Turkey; 3 Department of Ophthalmology, Eskişehir Osmangazi University, Eskisehir, Turkey

**Keywords:** Retinal diseases, Macular degeneration

## Abstract

**Objectives:**

To assess the long-term efficacy of intravitreal anti-vascular endothelial growth factor (anti-VEGF) therapy on best-corrected visual acuity (BCVA) and foveal morphology in patients with extrafoveal macular neovascularisation (MNV) secondary to age-related macular degeneration (AMD) over five years.

**Methods:**

A total of 104 eyes with treatment-naïve extrafoveal MNV treated with intravitreal anti-VEGF injections were analysed retrospectively. BCVA was assessed at baseline and annually for five years. Central foveal thickness (CFT), intraretinal fluid (IRF), subretinal fluid (SRF), pigment epithelial detachments (PED), subretinal hyperreflective material (SHRM), and foveal atrophy (incomplete/complete retinal pigment epithelium and outer retinal atrophy (iRORA/cRORA))—were documented.

**Results:**

After five years, 46% of the eyes had unchanged or improved vision by one or more lines, whereas mean BVCA declined from 0.28 ± 0.20 logMAR at baseline to 0.50 ± 0.49 logMAR after five years (*p* = 0.016). CFT, and the prevalence of IRF and SRF decreased significantly (*p* < 0.001), while iRORA (*p* = 0.041), and cRORA (*p* < 0.001) increased by year five. Presence of cRORA was associated with worse five-year BCVA (*p* < 0.001).

**Conclusion:**

Anti-VEGF therapy for extrafoveal MNV secondary to AMD stabilised or improved BCVA in approximately half of the patients; however, mean BCVA declined after five years. Long-term functional benefits were limited due to morphological changes in the macula, such as subfoveal atrophy.

## Introduction

Neovascular age-related macular degeneration (nAMD) is the leading cause of vision impairment in elderly individuals in industrialised countries, particularly when macular neovascularisation (MNV) involves the fovea [1–3]. MNV can result in exudation, subretinal haemorrhages, fibrosis, and macular atrophy resulting in progressive and irreversible vision loss at advanced stages.

Trials on photodynamic therapy (PDT) with verteporfin and intravitreal anti-vascular endothelial growth factor (VEGF) therapy demonstrated efficacy in managing subfoveal nAMD. However, no randomised controlled clinical trial has focused on extrafoveal MNV [4–12]. The Macular Photocoagulation Study (MPS) group conducted the only prospective randomised trial investigating the treatment effects of laser photocoagulation on extrafoveal MNVs during the 1980s and 1990s [13, 14]. These studies provide valuable insights but may now be outdated given the availability of anti-VEGF agents for intravitreal therapy.

Subfoveal and juxtafoveal MNV pose a significant threat to visual function due to their direct impact on foveal morphology. In contrast, eyes with extrafoveal MNV in nAMD, accounting for about 5–8% of all MNVs in nAMD, usually present with better visual acuity and may have a more favourable prognosis, provided the fovea remains unaffected or only minimally involved [15–18]. Despite anti-VEGF therapy now being the standard of care for MNV secondary to nAMD, PDT and laser photocoagulation remain rarely used alternatives for extrafoveal lesions [19–23]. However, robust data regarding the long-term effects of anti-VEGF therapy in extrafoveal MNV are scarce, leaving a significant gap in the current literature [24, 25]. This study aims to report the functional and morphological outcomes of intravitreal anti-VEGF therapy for extrafoveal MNV secondary to nAMD in a tertiary centre over a five-year period.

## Methods

### Study design and patient selection

This large retrospective study analysed data from treatment-naïve eyes with newly diagnosed, extrafoveal MNV secondary to nAMD [13, 26]. Patients were treated at the Department of Ophthalmology, University of Tübingen, Germany, between May 2009 and October 2019. Fluorescein angiography (FA) images from over 3500 consecutive patients with AMD were retrospectively reviewed to identify cases with extrafoveal MNV. A senior retina specialist (FG) assessed the type and localisation of the MNV in respect to the inclusion criteria. The inclusion criteria were: 1—age ≥50 years; 2—diagnosis of nAMD confirmed by clinical examination and ancillary imaging [26]; 3—extrafoveal MNV confirmed by FA; 4—follow-up of at five years; 5—available optical coherence tomography (OCT) at baseline and all follow-up examinations.

Exclusion criteria were: 1—subfoveal, juxtafoveal or juxtapapillary MNV l; 2—previous treatment of nAMD; 3—high myopia (≥ −6 dpt); 4—MNV secondary to conditions other than nAMD (e.g. high myopia, trauma, angioid streaks, central serous chorioretinopathy); 5—ocular comorbidities affecting the macular morphology (e.g., diabetic maculopathy, retinal vein occlusion, vitreomacular interface diseases); 6—polypoidal choroidal vasculopathy (PCV); 7—amblyopia; 8—previous retinal surgery.

**Fig. 1:**
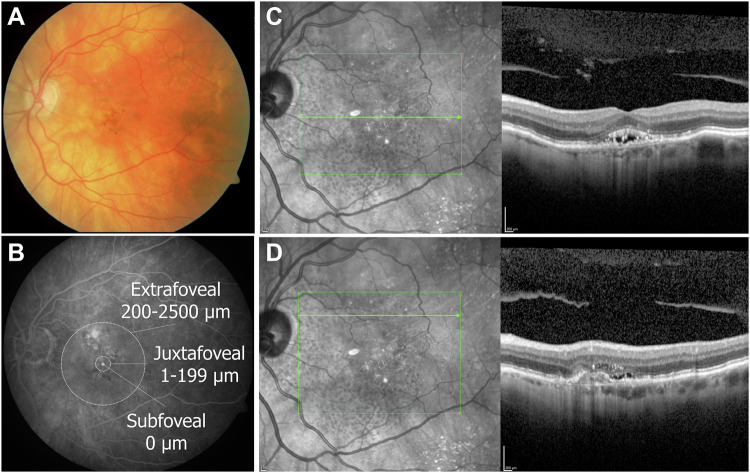
Multimodal Imaging of extrafoveal MNV. **A** Colour fundus photograph showing alterations in the retinal pigment epithelium and small haemorrhages. **B** Midphase of the fluorescein angiography delineates the extrafoveal localisation of the MNV superonasal to the fovea. **C** Horizontal OCT scan through the fovea shows subretinal fluid and intraretinal hyperreflective foci. **D** Horizontal OCT scan through the MNV lesion shows retinal thickening, RPE detachment, intra- and subretinal fluid, intra- and subretinal hyperreflective foci. MNV macular neovascularisation, RPE retinal pigment epithelium, FA fluorescein angiography, OCT optical coherence tomography.

All patients underwent comprehensive routine ophthalmological evaluations, including BCVA, slit-lamp biomicroscopy, dilated fundoscopy, and multimodal imaging (Fig. 1) using FA (digital fundus camera, Carl Zeiss Meditec AG, Jena, Germany), and OCT (SD-OCT; Spectralis, Heidelberg Engineering, Germany) imaging of the macula.

Extrafoveal MNV was defined as MNV with its border on the foveal side located between 200 µm and 2500 µm from the centre of the foveal avascular zone [13]. The distance of the foveal border of the MNV from the centre of the fovea was measured using FA imaging and corroborated with OCT data (Fig. 1). MNV subtypes (types 1, 2, or 3) were classified based on FA and OCT imaging.

BCVA was obtained as Snellen equivalent and converted to the logarithm of minimum angle of resolution (logMAR) for statistical analysis. Morphological characteristics assessed were central foveal thickness (CFT), intraretinal fluid (IRF), subretinal fluid (SRF), pigment epithelium detachment (PED), subretinal hyperreflective material (SHRM), and atrophy of the retinal pigment epithelium and outer retinal layer atrophy (RORA) within the central 400 µm subfield of the macula. Morphological criteria definitions of the Consensus Nomenclature for Reporting Neovascular Age-Related Macular Degeneration Data Study group were used [26]. Subfoveal atrophy was categorised as incomplete (iRORA) or complete (cRORA) based on specific OCT features [27, 28].

All patients received three monthly doses of intravitreal anti-VEGF therapy initially, followed by pro re nata (PRN) or treat and extend (TAE) strategy. The choice of anti-VEGF agent (ranibizumab 0.5 mg (Lucentis®, Novartis, Basel, Switzerland), aflibercept 2 mg (Eylea®, Bayer, Leverkusen, Germany), bevacizumab 1.25 mg (Avastin®, Roche, Basel, Switzerland)) and the therapy regimen after the upload phase (PRN or TAE) was at the discretion of the treating physician. This reflects real-world clinical practice and enhances the generalisability of the findings. A switch to an alternative anti-VEGF agent was performed in cases of suboptimal therapeutic response, as evidenced by persistent intraretinal and/or subretinal fluid on OCT. The number of intravitreal injections received by each patient over the five-year period was recorded.

### Statistical analysis

Statistical analyses were conducted using IBM SPSS Statistics for Windows, version 22.0 (IBM Corp, Armonk, NY). Normality of continuous variables was assessed using the Kolmogorov-Smirnov and Shapiro-Wilk tests. Categorical variables were expressed as frequency (n) and percentages (%), while continuous variables were expressed as mean (± standard deviation), median, and range (minimum-maximum). Chi Square, Cochran’s Q, and McNemar tests were used to detect changes in morphological features across the five years and between baseline and five years, respectively. The Kruskal-Wallis test was used to compare the number of injections from year to year. The Friedman test was employed to evaluate changes in BCVA and CFT over time. Post-hoc pairwise comparisons were adjusted using Bonferroni correction to control for multiple comparisons. Multiple linear regression models were applied to identify factors influencing BCVA at baseline and year five. Predictor variables included age, gender, baseline BCVA, CFT, and morphological features such as IRF, SRF, PED, SHRM, and iRORA/cRORA. The distance between the MNV’s posterior edge and the centre of the foveal avascular zone was also considered. A *p*-value of *p* ≤ 0.05 was considered statistically significant for all tests.

### Ethics approval

This study adhered to the tenets of the Declaration of Helsinki. Ethical approval was obtained from the institutional ethics committee of the University Hospital Tübingen before the analysis (project number: 626/2024BO2).

## Results

A total of 104 eyes of 93 treatment-naïve patients with extrafoveal MNV secondary to nAMD were included in this study. The mean ± SD age of the patients was 76.9 ± 6.6 years. The majority of patients (59.1%) were female. Among the included eyes, 52 (50.0%) were right eyes. A total of 82 patients (88.2%) presented with unilateral and 11 patients (11.8%) with bilateral extrafoveal MNV.

**Fig. 2:**
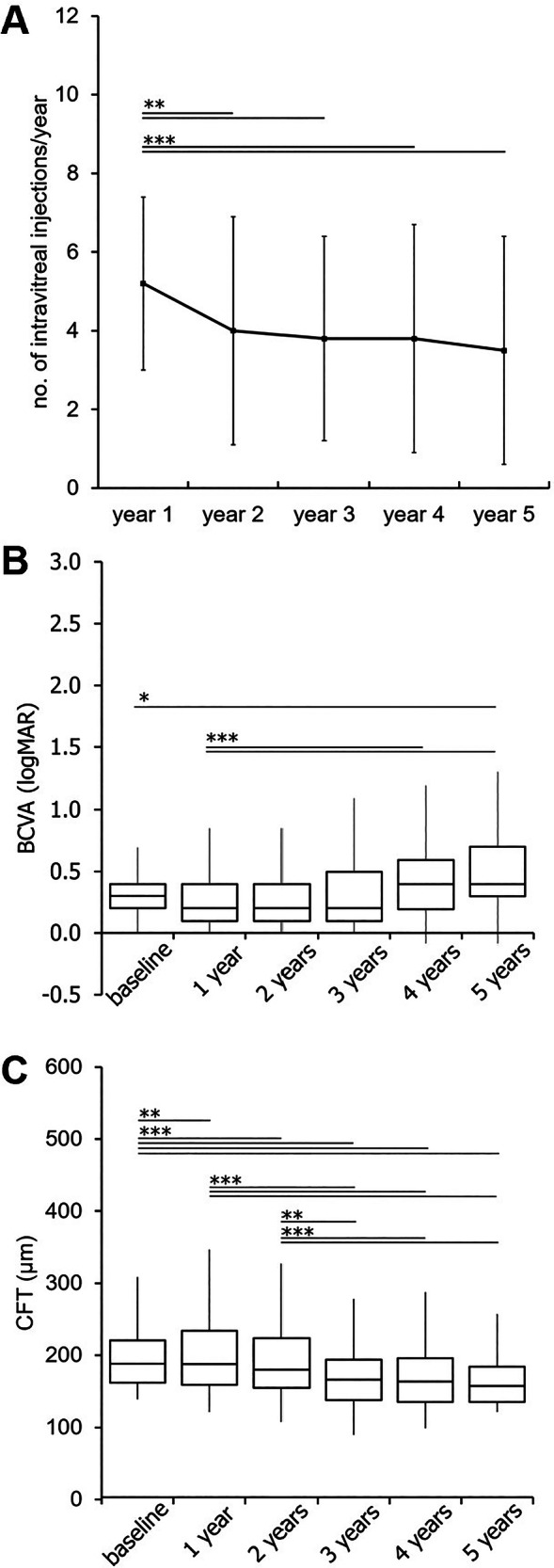
Treatment intensity, BCVA and CFT over the 5-year study period. **A** Mean number of intravitreal injections per year. Injection frequency peaked in year 1 and declined significantly thereafter, stabilising at approximately four injections per year. **B** Best-corrected visual acuity (BCVA, logMAR). A significant decline in BCVA was observed between baseline and five-year follow-up. **C** Central foveal thickness (CFT, µm). CFT significantly decreased within the first three years and remained stable thereafter. Values are presented as (**A**) mean ± standard deviation, and as (**B**, **C**) box-and-whisker plots, showing minimum, lower quartile, median, upper quartile, and maximum). Asterisks denote statistical significance: *p ≤ 0.05); **p ≤ 0.01); ***p ≤ 0.001).

At baseline, 69 eyes (66.3%) were phakic and 35 eyes (33.7%) pseudophakic, with the number of pseudophakic eyes increasing to 63 (60.6%) after five years. The mean distance of the foveal border of MNV from the centre of the foveal avascular zone was 787.4 ± 273.9 µm. Type 1 was the most common MNV subtype (62 eyes (59.6%)) followed by type 3 MNV (24 eyes (23.1%)) and type 2 MNV (18 eyes (17.3%)). Patients received a mean of 20.3 ± 10.5 (range: 3–57) anti-VEGF injections per eye over the five-year follow-up period. Bevacizumab was used in 57 eyes (54.8%) in the upload phase, ranibizumab in 39 eyes (37.5%) and aflibercept in 8 eyes (7.7%). The mean number of injections administered per year decreased significantly in the years following the first year of treatment (year one: 5.2 ± 2.2 injections; year two: 4.0 ± 2.9 injections (*p* = 0.006); year three: 3.8 ± 2.6 injections (*p* = 0.002); year four: 3.8 ± 2.9 injections (*p* < 0.001); year five: 3.5 ± 2.9 injections (*p* < 0.001)) (Fig. 2).

Mean baseline BCVA at baseline was 0.28 ± 0.20 logMAR (20/40 Snellen) and 0.50 (20/63 Snellen) (*p* = 0.016) after five years (Fig. 2). Compared to baseline, no significant change in mean BCVA was observed for the first four years. After five years 29 eyes (27.9%) had improved BCVA by one or more lines, 56 eyes (53.8%) experienced a decline, and 19 eyes (18.3%) maintained stable vision. Among the 56 eyes with a BCVA decline, 19.6% lost one line, 30.4 two lines, and 50.0% three or more lines.

Seventy-five eyes (72.1%) had a relatively good baseline BCVA of 0.3 logMAR (≥20/40 Snellen)—an important threshold for a good BCVA with legal and functional implications (in many countries minimum BCVA required to drive, required by several occupations, clinical benchmark to assess moderate visual impairment). Fourty-six eyes (41.8%) had a baseline BCVA of ≤0.2 logMAR (≥20/32 Snellen) and 25 eyes (24.0%) had a BCVA of 0.1 or 0.0. After five years only 36 eyes had a BCVA of ≤ 0.3.

A sub-analysis of BCVA in respect to the MNV subtypes revealed significantly worse mean baseline BCVA (0.39 ± 0.22 logMAR) for type 3 compared to type 1 (0.25 ± 0.20 logMAR, *p* = 0.020) and type 2 (0.23 ± 0.14 logMAR, *p* = 0.043). However, the mean BCVA at five years did not show significant differences between the MNV subtypes (*p* = 0.123) (Table 1). In MNV type 1, 30 eyes (48.4%) had stable or improved BCVA after five years, while 32 eyes (51.6%) decreased in BCVA. Type 2 was associated with a higher portion of eyes with BCVA decline than other MNV subtypes (BCVA stable/improved: 6 eyes (33.3%), BCVA decline: 12 eyes (66.6%)). Half of the patients (12 eyes (50.0%)) with type 3 MNV showed BCVA deterioration.

**Table 1:** Baseline and 5-year clinical and morphological characteristics of eyes with MNV types 1, 2, and 3. Data included BCVA, CFT, and prevalence of IRF, SRF, PED, SHRM, and atrophy (iRORA, cRORA).

MNV subtype	MNV type 1			MNV type 2			MNV type 3			Differences between MNV-types	Total		
**No. of eyes**, *n* (%)	62 (59.6%)			18 (17.3)			24 (23.1)				104 (100)		
***visit***	baseline	5 yrs	p	baseline	5 yrs	p	baseline	5 yrs	p	**p**^**baseline**^ **/ p**^**5yrs**^	baseline	5 yrs	p
**BCVA** (logMAR, mean ± SD)	0.25 ± 0.20	0.44 ± 0.48	**0.001**^**a**^	0.23 ± 0.14	0.57 ± 0.49	**0.007**^a^	0.39 ± 0.22	0.61 ± 0.52	0.109^a^	**0.013**^b^ / 0.123^b^	0.28 ± 0.20	0.50 ± 0.49	**0.016**^a^
**CFT** (µm, mean ± SD	244 ± 88	173 ± 87	**<0.001**^a^	255 ± 86	160 ± 45	**0.001**^a^	287 ± 101	151 ± 100	**<0.001**^a^	0.174^b^ / 0.063^b^	255 ± 91	165 ± 85	**<0.001**^a^
**IRF** (*n* (%))	8 (12.9)	5 (8.1)	0.549^c^	2 (11.1)	2 (11.1)	1.000^c^	19 (79.2)	6 (25.0)	**0.002**^c^	**<0.001 **^d^ / 0.101 ^d^	29 (27.9)	13 (12.5)	**0.006**^c^
**SRF** (*n* (%))	32 (51.6)	8 (12.9)	**<0.001**^c^	12 (66.7)	4 (22.2)	**0.021**^c^	8 (33.3)	0 (0)	**0.008**^c^	0.094 ^d^ / 0.072 ^d^	52 (50.0)	12 (11.5)	**<0.001**^c^
**PED**, (*n* (%))	7 (11.3)	6 (9.7)	1.000^c^	0 (0)	0 (0)	-	5 (20.8)	1 (4.2)	0.219^c^	0.112 ^d^ / 0.300 ^d^	12 (11.5)	7 (6.7)	0.383^c^
**SHRM**, (*n* (%))	12 (19.4)	15 (24.2)	0.607^c^	6 (33.3)	6 (33.3)	1.000^c^	2 (8.3)	7 (29.2)	0.125^c^	0.126 ^d^ / 0.715 ^d^	20 (19.2)	28 (26.9)	1.000^c^
**iRORA** (*n* (%))	3 (4.8)	8 (12.9)	0.182^c^	2 (11.1)	3 (16.7)	1.000^c^	1 (4.2)	5 (20.8)	0.219^c^	0.510^e^ / 0.646^e^	6 (5.8)	16 (15.4)	**0.041**^c^
**cRORA** (*n* (%))	2 (3.2)	13 (21.0)	**<0.003**^c^	0 (0)	6 (33.3)	**0.031**^c^	0 (0)	13 (30.8)	**<0.001**^c^	1.000^e^ / **0.014**^e^	2 (1.9)	32 (30.8)	**<0.001**^c^

*P*-values represent within-group comparisons over time, and between-group comparisons across MNV types.

Significant *p*-values are shown in bold.

*MNV* macular neovascularisation, *BCVA* best-corrected visual acuity, *CFT* central foveal thickness, *IRF* intraretinal fluid, *SRF* subretinal fluid, *PED* pigment epithelium detachment, *SHRM* subretinal hyperreflective material, *iRORA/cRORA* incomplete/complete retinal pigment epithelium and outer retinal atrophy.

^a^Wilcoxcon Signed Rank-Test.

^b^Kruskal-Wallis-Test.

^c^McNemar-test.

^d^Chi-squared test comparing differences in the prevalence of morphological characteristics between MNV subtypes at baseline (p^baseline^) or five year-examination (p^5yrs^).

^e^Fisher-Freeman-Halton Exact Test.

Mean CFT decreased significantly from 255.5 ± 91.2 µm at baseline to 213.6 ± 104.2 µm (*p* = 0.003) after the first year and to 165.7 ± 84.5 µm after five years (*p* < 0.001) (Fig. 2). Yearly data for BCVA and morphological parameters are provided in Supplemental Table 1.

**Fig. 3:**
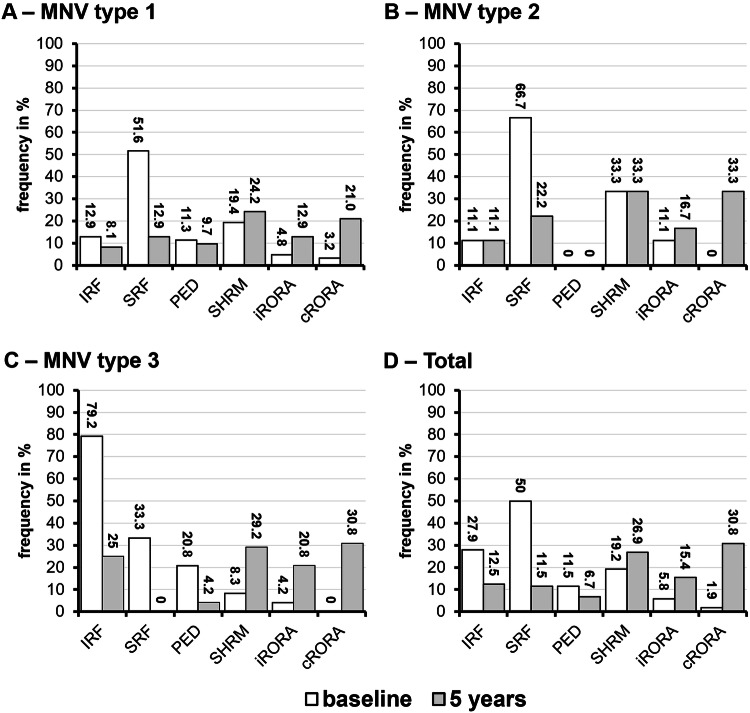
Morphological parameters at baseline (white bars) and after 5 years (grey bars) stratified by MNV subtype. **A** MNV type 1, **B** type 2, **C** type 3, and **D** all types combined. Assessed features include IRF, SRF, PED, SHRM, iRORA, and cRORA. MNV macular neovascularisation, IRF intraretinal fluid, SRF subretinal fluid, PED pigment epithelium detachment, SHRM subretinal hyperreflective material, iRORA/cRORA incomplete/complete retinal pigment epithelium and outer retinal atrophy.

Over the five-year follow-up period, the prevalence IRF decreased notably, from 27.9% at baseline to 12.5% by year five (*p* = 0.006), and likewise SRF showed a significant reduction, from 50.0% at baseline to 11.5% at year five (*p* < 0.001). The presence of PED remained stable over time, with no significant change observed across the five years (*p* = 0.267). Also, the prevalence of SHRM increased, but not significantly, from 19.2% at baseline to 26.9% at year five (*p* = 1.000). In contrast, subfoveal atrophy in terms of iRORA/cRORA was found at baseline in 5.8%/1.9% of the eyes and 15.4%/30.8% at the five-year follow-up (*p* = 0.041/*p* < 0.001) (Fig. 3).

Factors significantly associated with worse baseline BCVA included higher CFT (*p* = 0.013), presence of IRF (*p* = 0.037), SHRM (*p* = 0.030), and cRORA (*p* = 0.001). SRF, PED, iRORA and the distance of the MNV to the fovea did not have a significant impact on baseline BCVA.

**Fig. 4:**
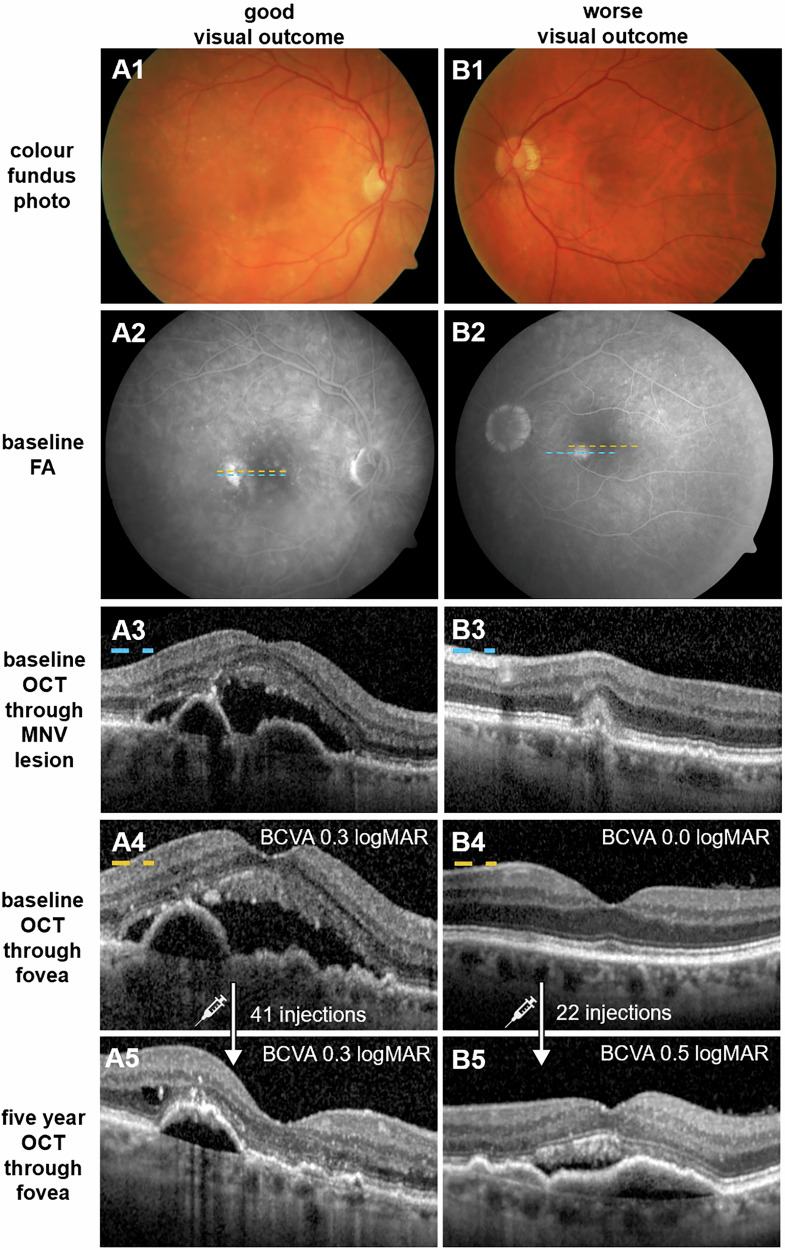
Representative cases of extrafoveal MNV treated with intravitreal anti-VEGF therapy. Good visual outcome (**A**): An 86-year-old female with MNV located temporal-inferior to the fovea in the right eye. The lesion’s central border was 751 µm from the foveal centre. Baseline BCVA of 0.3 logMAR (20/40 Snellen) was maintained over five years with 41 intravitreal anti-VEGF injections. Worse visual outcome (**B**): A 70-year-old male with MNV located nasal-inferior to the fovea in the left eye. The lesion’s central border was 775 µm from the foveal centre. Although initial BCVA was 0.0 logMAR (20/20 Snellen), it declined to 0.5 logMAR (20/63 Snellen) after five years despite 22 intravitreal injections. Panels show: A1/B1—colour fundus photography at baseline; A2/B2—FAat baseline; A3/B3—baseline OCT through the MNV lesion (blue dashed line in FA); A4/B4—baseline OCT through the fovea (yellow dashed line in FA); A5/B5—OCT through the fovea after five years of treatment. MNV macular neovascularisation, BCVA best-corrected visual acuity, FA fluorescein angiography, OCT optical coherence tomography.

Five-year mean BCVA was significantly associated with cRORA (*p* < 0.001), but not with iRORA (*p* = 0.612). SHRM was not significantly correlated with five-year BCVA (*p* = 0.215). None of the other morphological parameters showed a significant association with the mean BCVA at five-year (Fig. 4).

## Discussion

This study showed stabilisation or improvement of the BCVA in half of the patients with extrafoveal MNV secondary to AMD under anti-VEGF therapy after five years. However, the observed decline in mean BCVA in the whole cohort underlined the chronic, progressive nature of AMD despite ongoing treatment. The presented results align with prior studies, confirming the efficacy of anti-VEGF agents in managing exudation and delaying visual deterioration, but ultimately treatment cannot completely halt the progression of nAMD in the majority of patients [10, 25, 29–31].

Before the introduction of anti-VEGF therapies, thermal laser photocoagulation and PDT were the primary treatment alternatives for extrafoveal MNV. The Macular Photocoagulation Study (MPS) demonstrated that thermal laser photocoagulation reduces the risk of moderate and severe vision loss in patients with extrafoveal and juxtafoveal MNV by ablating lesions [14]. However, central scotomas caused by laser-induced retinal damage, and the high recurrence rate of MNV frequently led to subfoveal progression and limited the beneficial effect of this treatment [14, 32]. While PDT was initially promising in slowing MNV progression and stabilising vision, its long-term outcomes remained suboptimal [7, 21, 33]. PDT was unable to prevent significant functional decline and MNV recurrence in many patients [34]. Comparative studies confirmed that PDT was less effective than anti-VEGF therapy in preserving vision [7, 35, 36].

The introduction of anti-VEGF therapies revolutionised the management of nAMD. By inhibiting VEGF, these agents effectively reduce exudation and control disease activity without causing collateral damage to retinal structures, which was noted in thermal photocoagulation or PDT. Pivotal studies showed efficacy of anti-VEGF therapy in maintaining or improving vision in nAMD patients [7, 8, 11, 29, 37]. But, extrafoveal MNVs were not specifically addressed in these studies.

To our knowledge, there is only a single study that compared thermal laser photocoagulation of extrafoveal MNV with intravitreal anti-VEGF injections showing good efficacy of ranibizumab while laser photocoagulation was associated with visual acuity deterioration and high recurrence rates [38]. This case series included only 13 eyes in the subgroup treated with thermal laser photocoagulation and 11 eyes treated with intravitreal ranibizumab with a limited follow-up of 23.6 ± 2.26 and 19.1 ± 9.74 months, respectively. Other case series with smaller cohorts and shorter observation periods have reported the efficacy of anti-VEGF therapy in extrafoveal MNV, yielding results that align with our findings [24, 39]. Our data revealed preserved or improved vision after five years in a significant subset of eyes. However, mean BCVA decreased slowly in half of the patients under ongoing anti-VEGF therapy over the five-year period. These results reflect the heterogenous outcomes in our cohort.^2-4^

As expected, patients in our study had better baseline BCVA than those reported in studies that included subfoveal MNV [39, 40]. Therefore, the decline we observed may not only reflect poor treatment efficacy, but also the limited potential for improvement in patients who already start with higher baseline BCVA [41]. In fact, many eyes in our cohort showed relatively good baseline BCVA ((72.1% with BCVA of ≤ 0.3 logMAR (≥20/40 Snellen); 41.8% with ≤ 0.2 logMAR (≥20/32 Snellen); 24.0% with 0.1 logMAR (20/25 Snellen) or better)). Because of this “ceiling effect”, their capacity for further visual improvement was restricted. As a result, even modest disease progression or treatment limitations could lead to a proportionally larger decline in mean BCVA. In contrast, patients with poorer baseline BCVA often already have more advanced retinal damage or central involvement at therapy initiation, which limits the extent of further decline. In such cases, the modest functional gains from anti-VEGF treatment may counterbalance potential losses, resulting in relatively stable or slightly improved mean BCVA. These differences in baseline BCVA contribute to the variability in outcomes observed in our study.

The type of MNV was not found to affect the outcome after five years. Although MNV type 3 had worse baseline BCVA, the five-year BCVA did not differ between MNV subtypes. However, the percentage of eyes with a decline in BCVA differed between MNV types [42]. Subfoveal atrophy (cRORA) increased in all MNV subtypes making it the only morphological parameter that is associated with the 5-year BCVA outcome. A high increase in cRORA was seen in type 3 MNV from 0 eyes (0.0%) to 13 eyes (30.8%). Interpretation of morphological changes is limited by the relatively small sample size of eyes with MNV types 2 and 3.

This study’s strengths include a large cohort size and the extended follow-up duration, making it the most extensive study to date on the long-term outcomes of anti-VEGF therapy for treatment-naïve extrafoveal MNV in nAMD. However, the lack of standardised treatment protocols is a notable limitation. ICGA was performed on physician discretion in cases with suspected PCV or suboptimal response to therapy. Therefore, the presence of undetected PCV or pachychoroid neovascularisation in some eyes cannot be ruled out with certainty. Furthermore, the absence of a control group, while ethically understandable, limits comparison with alternative treatment modalities. Change of the lens status over the treatment period could also impact BCVA outcomes. However, our statistical analysis showed no significant differences in BCVA between phakic and pseudophakic patients at baseline or final follow-up. Prospective studies with standardised protocols would provide more definitive results, though the low prevalence of extrafoveal MNV presents challenges for patient recruitment. Moreover, morphological parameters were assessed on OCT scans. Autofluorescence imaging would improve the visibility of RPE-atrophy and enhance the analysis, which was not a standard procedure in this retrospective real-world study.

Our results emphasise the importance early detection and intervention for extrafoveal MNV, since baseline BCVA was a significant predictor of the long-term outcome. While anti-VEGF therapy effectively reduces the exudation and stabilises BCVA initially, its long-term functional benefits were limited for a number of patients, because of the morphological changes in the macula, such as atrophy. This highlights the need to optimise current treatment approaches, and to explore alternatives aiming to preserve the morphological integrity of the macula.

## Summary

### What was known before:

- Anti-VEGF therapy is effective for subfoveal MNV in AMD.

### What this study adds:

- This study reports the largest long-term real-world evaluation of anti-VEGF therapy for extrafoveal MNV secondary to AMD from a tertiary centre.
- Although best-corrected visual acuity (BCVA) remained stable or improved in one half of the eyes, the overall mean BCVA declined within five years. Morphologically, reduction in CFT and foveal fluid accumulation was seen, but subretinal hyperreflective material and atrophy increased.
- While anti-VEGF is beneficial, it cannot entirely halt the progression of visual loss in extrafoveal MNV secondary to AMD.

## Supplementary information

Supplemental Table 1

## Data Availability

The datasets generated and analysed during the current study are available from the corresponding author on reasonable request.

## References

[1] Wong WL, Su X, Li X, Cheung CMG, Klein R, Cheng CY, et al. Global prevalence of age-related macular degeneration and disease burden projection for 2020 and 2040: A systematic review and meta-analysis. Lancet Glob Heal. 2014;2:e106–16. 10.1016/S2214-109X(13)70145-1 25104651

[2] Colijn JM, Buitendijk GHS, Prokofyeva E, Alves D, Cachulo ML, Khawaja AP, et al. Prevalence of Age-Related Macular Degeneration in Europe: The Past and the Future. Ophthalmology. 2017;124:1753–63. 28712657 10.1016/j.ophtha.2017.05.035 PMC5755466

[3] Kawasaki R, Yasuda M, Song SJ, Chen SJ, Jonas JB, Wang JJ, et al. The prevalence of age-related macular degeneration in Asians: a systematic review and meta-analysis. Anal Ophthalmol. 2010;117:921–7. 10.1016/j.ophtha.2009.10.007 20110127

[4] Bressler NM. Photodynamic Therapy of Subfoveal Choroidal Neovascularization in Age-Related Macular Degeneration With Verteporfin. Arch Ophthalmol. 2001;119:198–207. 11176980

[5] Blumenkranz MS, Bressler NM, Bressler SB, Donati G, Fish GE, Haynes LA, et al. Verteporfin Therapy for Subfoveal Choroidal Neovascularization in Age-Related Macular Degeneration. Arch Ophthalmol. 2002;120:1307–14. 12365909 10.1001/archopht.120.10.1307

[6] Azab M, Boyer DS, Bressler NM, Bressler SB, Cihelkova I, Hao Y, et al. Verteporfin Therapy of Subfoveal Minimally Classic Choroidal Neovascularization in Age-Related Macular Degeneration. Arch Ophthal. 2015;123:448–57. 10.1001/archopht.123.4.448 15824216

[7] Brown DM, Michels M, Kaiser PK, Heier JS, Sy JP, Ianchulev T. Ranibizumab versus verteporfin photodynamic therapy for neovascular age-related macular degeneration: Two-year results of the ANCHOR study. Ophthalmology. 2009;116:57–65.e5. 19118696 10.1016/j.ophtha.2008.10.018

[8] Rosenfeld PJ, Brown DM, Heier JS, Boyer DS, Kaiser PK, Chung CY, et al. Ranibizumab for Neovascular Age-Related Macular Degeneration. N Engl J Med. 2006;355:1419–31. 17021318 10.1056/NEJMoa054481

[9] Heier JS, Brown DM, Chong V, Korobelnik J. Intravitreal Aflibercept (VEGF Trap-Eye) in Wet Age-Related Macular Degeneration. Ophthalmology. 2012;119:2537–48. 10.1016/j.ophtha.2012.09.006 23084240

[10] Maguire MG, Martin DF, Ying G, Jaffe GJ, Daniel E, Grunwald JE, et al. Five-Year Outcomes with Anti–Vascular Endothelial Growth Factor Treatment of Neovascular Age-Related Macular Degeneration: The Comparison of Age-Related Macular Degeneration Treatments Trials. Ophthalmology. 2016;123:1751–61. 27156698 10.1016/j.ophtha.2016.03.045 PMC4958614

[11] Schmidt-Erfurth U, Kaiser PK, Korobelnik J, Brown DM, Chong V, Nguyen QD, et al. Intravitreal Aflibercept Injection for Neovascular Age-related Macular Degeneration Ninety-Six-Week Results of the VIEW Studies. Ophthalmology. 2014;121:193–201. 24084500 10.1016/j.ophtha.2013.08.011

[12] Dugel PU, Koh A, Ogura Y, Jaffe GJ, Schmidt-Erfurth U, Brown DM, et al. HAWK and HARRIER: Phase 3, Multicenter, Randomized, Double-Masked Trials of Brolucizumab for Neovascular Age-Related Macular Degeneration. Ophthalmology. 2020;127:72–84. 30986442 10.1016/j.ophtha.2019.04.017

[13] Macular Photocoagulation Study Group. Argon Laser Photocoagulation for Senile Macular Degeneration. Results of a Randomized Clinical Trial. Arch Ophthalmol. 1982;100:912–18. 10.1001/archopht.1982.01030030920003 7046707

[14] Macular Photocoagulation Study Group. Argon Laser Photocoagulation for Neovascular Maculopathy: Five-Year Results From Randomized Clinical Trials. Arch Ophthalmol. 1991;109:1109–14. 1714270

[15] Olsen TW, Feng X, Kasper TJ, Rath PP, Steuer ER. Fluorescein angiographic lesion type frequency in neovascular Age-Related macular degeneration. Ophthalmology. 2004;111:250–5. 15019371 10.1016/j.ophtha.2003.05.030

[16] George S, Cooke C, Chakravarthy U. Exudative AMD subtypes and eligibility for treatment with ranibizumab. Eye. 2010;24:1247–51. 20019764 10.1038/eye.2009.301

[17] Cohen SY, Creuzot-Garcher C, Darmon J, Desmettre T, Korobelnik JF, Levrat F, et al. Types of choroidal neovascularisation in newly diagnosed exudative age-related macular degeneration. Br J Ophthalmol. 2007;91:1173–6. 17383997 10.1136/bjo.2007.115501 PMC1954889

[18] Beaumont PE, Kang HK. Lesion morphology in age-related macular degeneration and its therapeutic significance. Arch Ophthalmol. 2006;7:214–5. 10.1001/archopht.124.6.807 16769834

[19] Rosenblatt BJ, Shah GK, Blinder K. Photodynamic therapy with verteporfin for peripapillary. Retina. 2005;25:33–7. 15655438 10.1097/00006982-200501000-00004

[20] Spaide RF, Sorenson J, Maranan L. Combined photodynamic therapy and intravitreal triamcinolone for nonsubfoveal choroidal neovascularization. Retina. 2005;6:685–90. 10.1097/00006982-200509000-00001 16141854

[21] Voelker M, Gelisken F, Ziemssen F, Wachtlin J, Grisanti S. Verteporfin photodynamic therapy for extrafoveal choroidal neovascularisation secondary to age-related macular degeneration. Graefe’s Arch Clin Exp Ophthalmol. 2005;243:1241–6. 16010552 10.1007/s00417-005-0021-8

[22] Barbara SH, Fine SL, Gass JDM. Macular Photocoagulation Study Group. Argon laser photocoagulation for neovascular maculopathy. Three-year results from randomized clinical trials. Arch Ophthalmol. 1986;104:694–701. 2423061

[23] Han DP, McAllister JT, Weinberg DV, Kim JE, Wirostko WJ. Combined intravitreal anti-VEGF and verteporfin photodynamic therapy for juxtafoveal and extrafoveal choroidal neovascularization as an alternative to laser photocoagulation. Eye. 2010;24:713–6. 19498454 10.1038/eye.2009.122

[24] Parodi M, Iacono P, Spina C, Iuliano L, Giudice G, Introini U, et al. Intravitreal ranibizumab for naive extrafoveal choroidal neovascularization secondary to age-related macular degeneration. Retina. 2014;34:2167–70. 24999724 10.1097/IAE.0000000000000223

[25] Pauleikhoff D, Gunnemann ML, Ziegler M, Heimes-Bussmann B, Bormann E, Bachmeier I, et al. Morphological changes of macular neovascularization during long-term anti-VEGF-therapy in neovascular age-related macular degeneration. PLoS One. 2023;18:e0288861. 38134207 10.1371/journal.pone.0288861 PMC10745158

[26] Spaide RF, Jaffe GJ, Sarraf D, Freund KB, Sadda SR, Staurenghi G, et al. Consensus Nomenclature for Reporting Neovascular Age-Related Macular Degeneration Data: Consensus on Neovascular Age-Related Macular Degeneration Nomenclature Study Group. Ophthalmology. 2020;127:616–36. 31864668 10.1016/j.ophtha.2019.11.004 PMC11559632

[27] Sadda SR, Guymer R, Holz FG, Schmitz-Valckenberg S, Curcio CA, Bird AC, et al. Consensus Definition for Atrophy Associated with Age-Related Macular Degeneration on OCT Classification of Atrophy Report 3. Ophthalmology. 2018;125:537–48. 29103793 10.1016/j.ophtha.2017.09.028 PMC11366072

[28] Guymer RH, Rosenfeld PJ, Curcio CA, Holz FG, Staurenghi G, Freund KB, et al. Incomplete Retinal Pigment Epithelial and Outer Retinal Atrophy in Age-Related Macular Degeneration. Ophthalmology. 2020;127:394–409. 31708275 10.1016/j.ophtha.2019.09.035 PMC7218279

[29] Rofagha S, Bhisitkul RB, Boyer DS, Sadda SR, Zhang K, SEVEN-UP Study Group. Seven-year outcomes in ranibizumab-treated patients in ANCHOR, MARINA, and HORIZON A multicenter cohort study (SEVEN-UP). Ophthalmology. 2013;120:2292–9. 10.1016/j.ophtha.2013.03.046 23642856

[30] Daniel E, Toth CA, Grunwald JE, Jaffe GJ, Martin DF, Fine SL, et al. Risk of Scar in the Comparison of Age-related Macular Degeneration Treatments Trials. Ophthalmology. 2014;121:656–66. 24314839 10.1016/j.ophtha.2013.10.019 PMC3943618

[31] Grunwald JE, Daniel E, Huang J, Ying G, Maguire MG, Toth CA, et al. Risk of Geographic Atrophy in the Comparison of Age-related Macular Degeneration Treatments Trials. Ophthalmology. 2014;121:150–61. 24084496 10.1016/j.ophtha.2013.08.015 PMC3892560

[32] Macular Photocoagulation Study Group. Persistent and Recurrent Neovascularization After Krypton Laser Photocoagulation for Neovascular Lesions of Age-Related Macular Degeneration. Arch Ophthal. 1990;108:825–31. 1693497 10.1001/archopht.1990.01070080067037

[33] Wachtlin J, Stroux A, Wehner A, Heimann H, Forster MH. Photodynamic therapy with verteporfin for choroidal neovascularisations in clinical routine outside the TAP study. One- and two-year results including juxtafoveal and extrafoveal CNV. Graefes Arch Clin Exp Ophthalmol. 2005;243:438–45. 15672299 10.1007/s00417-004-1071-z

[34] Potter MJ, Szabo SM. Recurrence of choroidal neovascularisation after photodynamic therapy in patients with age-related macular degeneration. Br J Ophthalmol. 2007;91:753–7. 17229805 10.1136/bjo.2006.110239 PMC1955583

[35] Larsen M, Schmidt-Erfurth U, Lanzetta P, Wolf S, Simader C, Tokaji E, et al. Verteporfin plus Ranibizumab for Choroidal Neovascularization in Age-related Macular Degeneration. Ophthalmology. 2012;119:992–1000. 22424834 10.1016/j.ophtha.2012.02.002

[36] Brown DM, Kaiser PK, Michels M, Soubrane G, Heier JS, Kim RY, et al. Ranibizumab versus Verteporfin for Neovascular Age-Related Macular Degeneration. N Engl J Med. 2006;355:1432–44. 17021319 10.1056/NEJMoa062655

[37] CATT Research Group, Martin DF, Maguire MG, Ying GS, Grunwald JE, Fine S. Ranibizumab and Bevacizumab for Neovascular Age-Related Macular Degeneration. N Engl J Med. 2011;364:1897–908. 21526923 10.1056/NEJMoa1102673 PMC3157322

[38] Ladas ID, Chatziralli P, Kotsolis AI, Douvali M, Georgalas I, Theodossiadis PG, et al. Intravitreal Ranibizumab versus Thermal Laser Photocoagulation in the Treatment of Extrafoveal Classic Choroidal Neovascularization secondary to Age-Related Macular Degeneration. Ophthalmologica. 2012;228:93–101. 10.1159/000337347 22571933

[39] Singer MA, Awh CC, Sadda S, Freeman WR, Antoszyk AN, Wong P, et al. HORIZON: An Open-Label Extension Trial of Ranibizumab for Choroidal Neovascularization Secondary to Age-Related Macular Degeneration. Ophthalmology. 2012;119:1175–83. 22306121 10.1016/j.ophtha.2011.12.016

[40] Chandra S, Rasheed R, Menon D, Patrao N, Lamin A, Gurudas S, et al. Impact of injection frequency on 5-year real-world visual acuity outcomes of a fl ibercept therapy for neovascular age-related macular degeneration. Eye. 2021;35:409–17. 32265509 10.1038/s41433-020-0851-y PMC8026582

[41] Ciulla TA, Hussain RM, Taraborelli D, Pollack JS, Williams DF. Longer-Term Anti-VEGF Therapy Outcomes in Neovascular Age-Related Macular Degeneration, Diabetic Macular Edema, and Vein Occlusion-Related Macular Edema: Clinical Outcomes in 130 247 Eyes. Ophthalmol Retin. 2022;6:796–806. 10.1016/j.oret.2022.03.021 35381391

[42] Mathis T, Holz FG, Sivaprasad S, Yoon YH, Eter N, Chen L, et al. Characterisation of macular neovascularisation subtypes in age-related macular degeneration to optimise treatment outcomes. 2023;37:1758–65. 10.1038/s41433-022-02231-y PMC10275926 36104522

