# Supplementary material for: Intravitreal anti-VEGF therapy for extrafoveal macular neovascularisation secondary to age-related macular degeneration: five-year results in a tertiary centre
Source: Eye (Lond). 2025 Nov 6;40(1):83–90. doi: 10.1038/s41433-025-04057-w (PMC12764817; doi:10.1038/s41433-025-04057-w)
Supplement: Supplementary file 1 — Supplemental Table 1 [file 41433_2025_4057_MOESM1_ESM.docx]

**Supplemental Table 1**

| **Characteristic** | **Baseline** | **1 year** | **2 years** | **3 years** | **4 years** | **5 years** |
| --- | --- | --- | --- | --- | --- | --- |
| BCVA, mean ± SD, logMAR | 0.28 ± 0.20 | 0.27 ± 0.23 | 0.38 ± 0.42 | 0.40 ± 0.42 | 0.43 ± 0.43 | 0.50 ± 0.49 |
| CFT, mean ± SD, µm | 255 ± 91 | 213± 104 | 198 ± 80 | 177 ± 75 | 170 ± 63 | 165 ± 85 |
| IRF, n (%) | 29 (27.9) | 14 (13.5) | 15 (14.4) | 12 (11.5) | 15 (14.4) | 13 (12.5) |
| SRF, n (%) | 52 (50.0) | 25 (24.0) | 26 (25.0) | 12 (11.5) | 19 (18.3) | 12 (11.5) |
| PED, n (%) | 12 (11.5) | 11 (10.6) | 11 (10.6) | 8 (7.7) | 6 (5.8) | 7 (6.7) |
| SHRM, n (%) | 20 (19.2) | 12 (11.5) | 19 (18.3) | 20 (19.2) | 25 (24.0) | 28 (26.9) |
| RORA, n (%)   - iRORA, n (%) - cRORA, n (%) | 8 (7.7)  6 (5.8)  2 (1.9) | 17 (16.4)  8 (7.7)  9 (8.7) | 23 (22.1)  11 (16.6)  12 (11.5) | 32 (30.8)  14 (13.5)  18 (17.3) | 42 (40.4)  18 (17.3)  24 (23.1) | 48 (46.2)  16 (15.4)  32 (30.8) |

Best-corrected visual acuity and morphological features at baseline and yearly follow-up examinations. BCVA: Best-corrected visual acuity; SD: standard deviation; CFT: central foveal thickness; SRF: subretinal fluid; IRF: intraretinal fluid; PED: pigment epithelial detachment; SHRM: subretinal hyperreflective material; iRORA/cRORA: incomplete/complete retinal pigment epithelium and outer retinal atrophy.
